# Subcellular Localization of Cytoplasmic Lattice-Associated Proteins Is Dependent upon Fixation and Processing Procedures

**DOI:** 10.1371/journal.pone.0017226

**Published:** 2011-02-16

**Authors:** Eric Morency, Lynne Anguish, Scott Coonrod

**Affiliations:** Baker Institute for Animal Health, College of Veterinary Medicine, Cornell University, Ithaca, New York, United States of America; McGill University, Canada

## Abstract

We and others have recently demonstrated by immuno-EM and mutation analysis that two oocyte-restricted maternal effect genes, PADI6 and MATER, localize, in part, to the oocyte cytoplasmic lattices (CPLs). During these ongoing studies, however, we found that the localization of these factors by confocal immunofluorescence (IF) analysis can vary dramatically depending upon how the oocytes and embryos are processed, with the localization pattern sometimes appearing more uniformly cytoplasmic while at other times appearing to be primarily cortical. We set out to better understand this differential staining pattern by testing a range of IF protocol parameters, changing mainly time and temperature conditions of the primary antibody solution incubation, as well as fixation methods. We found by confocal IF whole mount analysis that PADI6 and MATER localization in germinal vesicle stage oocytes is mainly cytoplasmic when the oocytes are fixed and then incubated with primary antibodies at room temperature for 1 hour, while the localization of these factors is largely limited to the cortex when the oocytes are fixed and incubated in primary antibody at 4°C overnight. We then probed sections of fixed/embedded ovaries and isolated two-cell embryos with specific antibodies and found that, under these conditions, PADI6 and MATER were again primarily cytoplasmically localized, although the staining for these factors is slightly more cortical at the two-cell stage. Taken together, our results suggest that the localization of CPL-associated proteins by confocal IF is particularly affected by processing conditions. Further, based on our current observations, it appears that PADI6 and MATER are primarily distributed throughout the cytoplasm as opposed to the oocyte subcortex.

## Introduction

Several years ago, we cloned and characterized peptidylarginine deiminase 6 (PADI6) from the oocyte proteome, based on its abundance and tissue-restricted expression pattern [1]. We then utilized whole mount confocal immunofluorescence (IF) microscopy to demonstrate that this maternal effect protein primarily localized throughout the egg and early embryo cytoplasm. Additionally, we also carried out immuno-electron microscopy analysis and found that, at the ultrastructural level, PADI6 primarily localized to the oocyte cytoplasmic lattices (CPLs). In subsequent reports, we utilized PADI6-null mice to document the requirement for PADI6 in CPL formation and for early cleavage divisions, thus highlighting the importance of PADI6 and the CPLs in early development [2], [3]. In these more recent studies, however, our confocal IF analysis found that PADI6 appeared to be much more cortically localized than we had previously observed. This cortical localization pattern was difficult to resolve in light of our immuno-EM data showing that PADI6 strongly localized to the CPLs throughout the cytoplasm and did not appear to be concentrated at the oocyte cortex. While we could not fully account for these differences, we predicted at the time that they were likely due to day-to-day variations in oocyte processing techniques.

MATER (NALP5) represents another oocyte-abundant protein that has been found by mutational analysis to be required for development beyond the two-cell stage [4]. While the function of this protein is not known, we have recently demonstrated that MATER localizes to the oocyte CPLs and that MATER is required for CPL formation [5]. The localization of MATER to the lattices has also been confirmed by other investigators [6]. Interestingly, MATER has also been identified as a member of the Sub Cortical Maternal Complex (SCMC) that also includes the maternal effect genes, Filia, Floped, and TLE6 [7]. This structure, as the name implies, has been localized to the oocyte and early embryo subcortex by confocal IF and this localization pattern seems somewhat at odds with the localization of MATER to the lattices throughout the cytoplasm. However, similar to PADI6, a review of previous MATER publications finds that the localization of MATER by confocal IF can vary considerably between studies from either primarily cortical to largely cytoplasmic [7], [8], [9], [10].

Taken together, these observations suggest that CPL associated proteins, such as MATER and PADI6, may be particularly sensitive to changes in processing and fixation conditions and thus their localization patterns can vary from study to study when confocal IF is used. In this report we tested this hypothesis and found that, when evaluating the subcellular distribution of PADI6 and MATER in isolated whole mount oocytes, changes in primary antibody incubation conditions and the type of fixative used, can greatly alter the cortical versus cytoplasmic distribution of these proteins. To support the hypothesis that these effects are primarily limited to CPL-associated proteins, we found that the localization of another oocyte-abundant protein, MSY2 [11], which does not colocalize well with PADI6, did not appear to be as strongly affected by these different processing procedures. Additionally, we show by IF analysis of tissue sections that, under these conditions, PADI6 and MATER are primarily distributed throughout the cytoplasm and are not concentrated at the cortex. These findings help to resolve previous inconsistencies regarding the subcellular localization of PADI6 and MATER, and suggest that these factors are primarily localized throughout the cytoplasm of the oocyte and early embryo. Finally, we hope that these new findings will provide technical insight for future investigations into the subcellular localization of egg factors within the cytoplasm of the oocyte and early embryo.

## Results

### Time and temperature influence the staining pattern of PADI6 and MATER, but not MSY2

**Figure 1:**
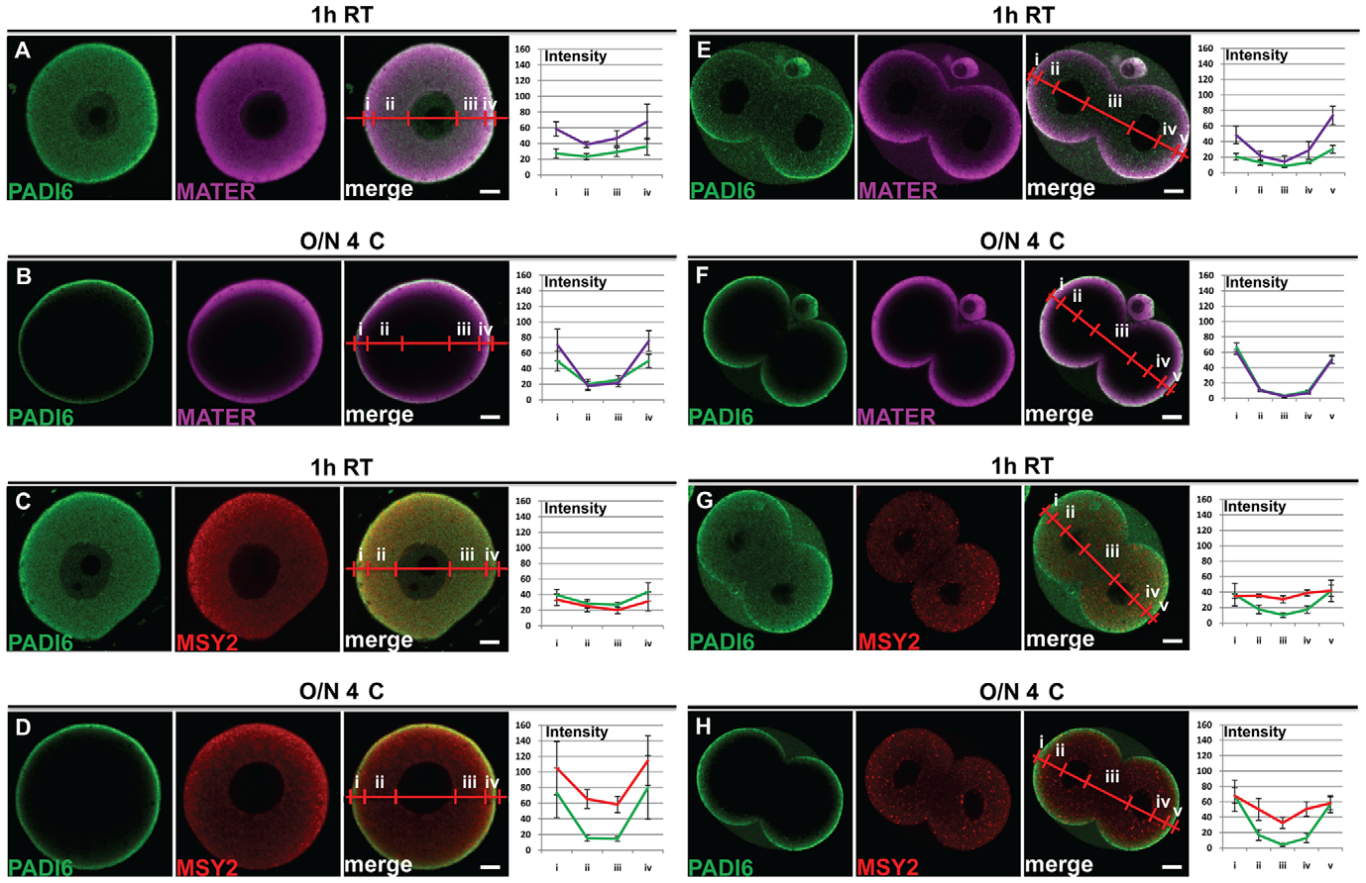
Time and temperature influence the staining pattern of PADI6 and MATER, but not MSY2. GV oocytes (A-D) and two-cell embryos (E-H) were prepared for IF and stained with antibodies against PADI6 (A-H), MATER (A, B, E and F) or MSY2 (C, D, G and H). Primary antibody incubation was carried out at either 1 h RT (A, C, E and G) or O/N 4°C (B, D, F and H). PADI6 is shown in green, MATER in magenta and MSY2 in red. Included are graphs of the averages and standard deviations of four intensity profiles of four images per condition of the different stains in either cortical (i and iv) or cytosolic (ii and iii) zones for the oocytes or cortical (i and v), basolateral cytosolic (iii) or apical cytosolic (ii and iv) zones for the embryos. Bars, 10 µm.

Our initial whole mount confocal IF study on the subcellular localization of PADI6 found that this factor is primarily localized throughout the oocyte cytoplasm [1], while in our more recent study we observed that PADI6 appears to be more cortically localized [3]. Comparison of the methods used for these studies found that, in the first study, we incubated oocytes and embryos in primary antibody for 1 h at room temperature (1 h RT), while in the subsequent study, primary antibody incubations were carried out overnight at 4°C (O/N 4°C). Given these differing localization patterns, we first directly tested whether primary antibody incubation time and temperature affected PADI6, MATER and MSY2 subcellular localization. Fully grown GV stage oocytes were isolated from ovarian follicles. Following fixation in paraformaldehyde (PFA) and subsequent permeabilization with Triton X-100, the oocytes were separated into two groups and incubated either 1 h RT or O/N 4°C in a primary antibody solution containing either anti-PADI6 and anti-MSY2, or anti-PADI6 and anti-MATER antibodies. Next, they were incubated with the secondary antibodies, washed, and mounted for visualization via confocal microscopy. The results are shown in figure 1. A distinct difference in the staining pattern for PADI6 and MATER was observed between the 1 h RT and O/N 4°C treatments (compare fig. 1A to 1B). Indeed, the cytoplasmic staining closest to the nucleus is much less apparent for PADI6 and MATER in the O/N 4°C condition, as well as there being an exacerbation of the cortical staining just under the cytoplasmic membrane compared to the 1 h RT condition. This is also evident when comparing the average intensity profiles of each image (compare fig. 1A to 1B), where the intensity of signal for PADI6 and MATER is very high in the cortex (sections i & iv), but extremely low in the cytosol (sections ii & iii) for the O/N 4°C condition compared to the 1 h RT condition. Furthermore, the difference of intensity between the cortical and cytoplasmic signal in the 1 h RT condition is not statistically significant (PADI6: p = 0.403; MATER: p = 0.08; MSY2: p = 0.066) while it is for the O/N 4°C condition (PADI6: p = 0.01; MATER: p = 0.004; MSY2: p = 0.015). In the case of MSY2, although the difference in staining intensity may be significant in the O/N 4°C condition the cytosolic signal is still readily visible compared to PADI6 (compare fig. 1C to 1D), indicating that not all proteins are subject to this effect to the same extent as PADI6 and MATER. Supplementary experiments with primary antibody incubations done either at 1 h 4°C or O/N RT suggested that time of primary antibody incubation affects the staining pattern more strongly than does temperature (fig. S1). Next, we tested whether changes in primary antibody incubation time and temperature also affected the localization of PADI6 and MATER in early cleavage-stage embryos. Therefore two-cell embryos were collected and processed similar to that described above for oocytes and imaged. Results (fig. 1E-H) show that, similar to GV-stage oocytes, the cytoplasmic staining of PADI6 and MATER is much less visible in the O/N 4°C condition compared to the 1 h RT condition and the cortical staining is much more pronounced (compare fig. 1E to 1F). This is especially visible on the intensity profiles where the signals for PADI6 and MATER between the two nuclei (section iii) are almost absent and much more elevated in the cortex (sections i & v) for the O/N 4°C condition. However, as opposed to oocytes, a stronger cortical signal is observed for both PADI6 and MATER in the 1 h RT primary antibody treatment group, even though the p values indicate that the difference between cortical and cytoplasmic staining is not significant for the 1 h RT condition (PADI6: p = 0.125; MATER: p = 0.11; MSY2: p = 0.79) whereas it is for the O/N 4°C for PADI6 and MATER but not MSY2 (PADI6: p = 0.025; MATER: p = 0.013; MSY2: p = 0.124); whose staining pattern is only slightly affected by the different incubation conditions in two-cell embryos. As a further control, we verified that two other components of oocytes were also unaffected by these two incubation conditions. Lipid droplets (cytoplasmic) and microtubules (mainly cortical) do not show a strong difference in localization between the 1 h RT and O/N 4°C incubation times (fig. S2).

### Different permeabilization conditions do not rescue the cytoplasmic staining of PADI6

**Figure 2:**
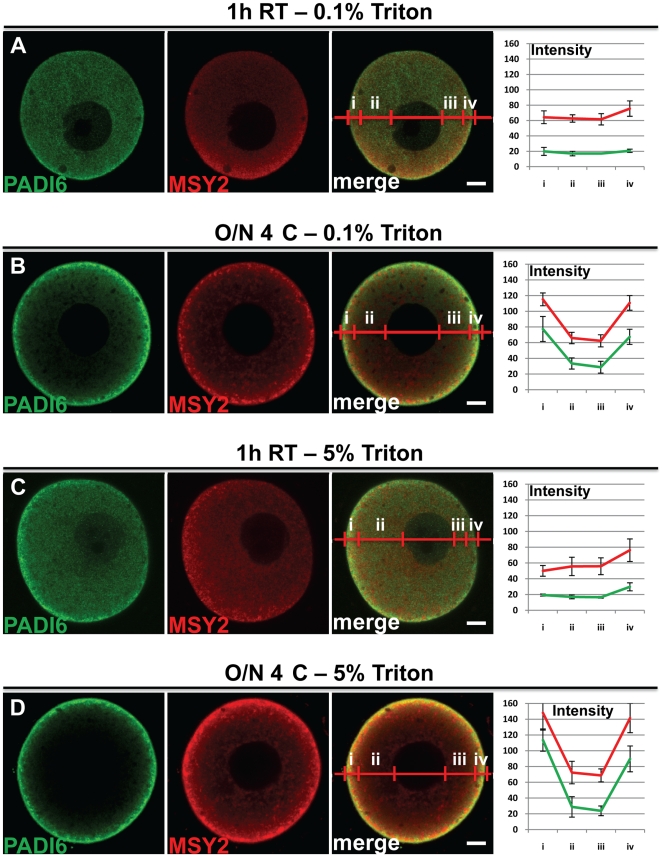
Different permeabilization conditions do not rescue the cytoplasmic staining of PADI6. GV oocytes were prepared for IF with either 0.1% (A and B) or 5% (C and D) Triton X-100 concentration in the permeabilization solution instead of the normal 0.5% concentration. Primary antibody incubation was carried out at either 1 h RT (A and C) or O/N 4°C (B and D). PADI6 is shown in green and MSY2 is shown in red. Included are graphs of the averages and standard deviations of four intensity profiles of four images per condition of the different stains in either cortical (i and iv) or cytosolic (ii and iii) zones. Bars, 10 µm.

The above results indicate that primary antibody incubation time, and to a lesser extent temperature, can influence the staining pattern of CPL-associated proteins, with longer incubation times leading to a stronger cortical signal. One possible explanation for the observed strong cortical PADI6 and MATER signal in the O/N 4°C incubation group is that the primary antibodies cannot fully penetrate the oocyte plasma membrane and, thus, cannot penetrate into the deeper cytoplasm. Alternatively, it is also possible that fixation with 4% PFA does not fully fix some proteins and, therefore, during the extended primary antibody incubation period, either the proteins or the protein-antibody complex may be released from their correct location and “drift” to the oocyte cortex. To test this hypothesis, we performed IF experiments on oocytes using two different concentrations of Triton X-100, 0.1% (fig. 2A and B) and 5% (fig. 2C and D) followed by either the 1 h RT or O/N 4°C primary antibody incubation. Given that increasing concentrations of Triton X-100 enhance cell membrane permeabilization [12], we reasoned that higher Triton X-100 concentrations would show an overall decrease in cortical staining, due to either enhanced antibody uptake or increased release of antigen antibody complexes. However, as shown in figure 2, we found that increasing concentrations of Triton X-100 had little effect on the PADI6 cortical signal in the O/N 4°C incubation group. These results suggest that the elevated PADI6 cortical signal is not due to effects associated with plasma membrane permeability. However, these experiments do not rule out the possibility that another structure at the oocyte cortex is affecting antibody permeability.

### Formalin fixation decreases the differential incubation effect on PADI6 and MATER localization

**Figure 3:**
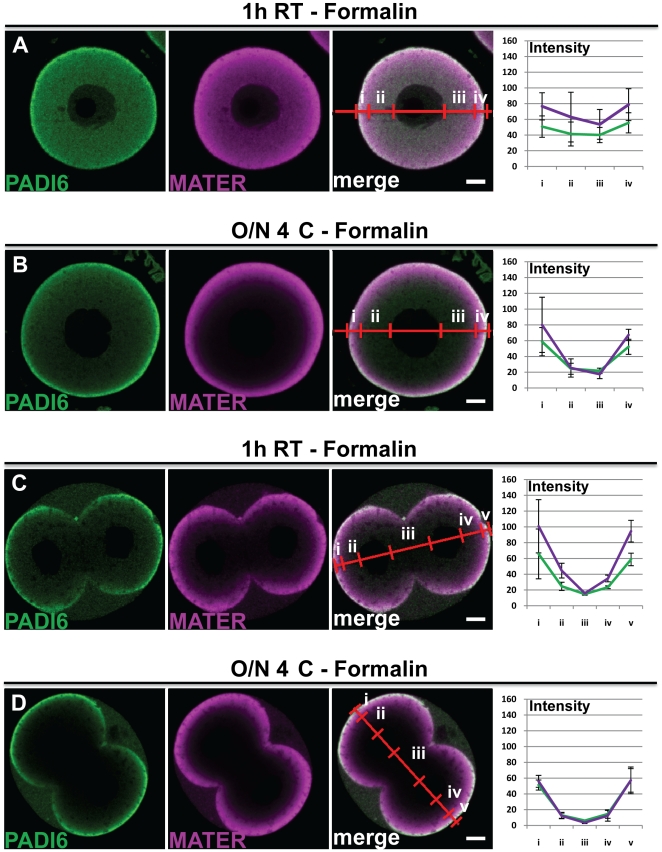
Formalin fixation decreases the differential incubation effect on PADI6 and MATER localization. GV oocytes (A and B) and two-cell embryos (C and D) were fixed with 10% formalin, prepared for IF and stained with antibodies against PADI6 and MATER (A-D). Primary antibody incubation was carried out at either 1 h RT (A and C) or O/N 4°C (B and D). PADI6 is shown in green and MATER in magenta. Included are graphs of the averages and standard deviations of four intensity profiles of four images per condition of the different stains in either cortical (i and iv) or cytosolic (ii and iii) zones for the oocytes or cortical (i and v), basolateral cytosolic (iii) or apical cytosolic (ii and iv) zones for the embryos. Bars, 10 µm.

Next, we more directly tested the hypothesis that a 30 minute 4% PFA treatment was insufficient for complete fixation of oocyte proteins. Thus, we performed similar experiments as in figure 1 but this time fixed the oocytes and embryos in a stronger fixative, 10% neutral buffered formalin, which contains ∼1.5% methanol as a stabilizing agent, and which is also a known precipitating fixative (versus solely cross-linking fixation with PFA alone) (fig. 3A, B, C and D). Results showed that the staining pattern of PADI6 and MATER in the oocytes and embryos is identical when fixed either in formaldehyde or formalin for the 1 h RT incubation condition (compare fig. 3A to 1A and fig. 3C to 1E respectively). However, in the O/N 4°C incubation condition, the cytoplasmic staining of PADI6 is better retained when using formalin fixation versus PFA (compare fig. 3B to 1B and fig. 3D to 1F respectively). As for MATER, there is more cytoplasmic staining visible in the formalin fixation condition, although the signal rescue is not as strong as with PADI6. These results indicate that, under certain conditions, formalin fixation appears to better preserve the localization pattern for PADI6, and to a lesser extent MATER.

### Analysis of PADI6 and MATER localization in embedded ovarian and oviduct cross sections reveals that these factors appear to be primarily localized throughout the cytoplasm

**Figure 4:**
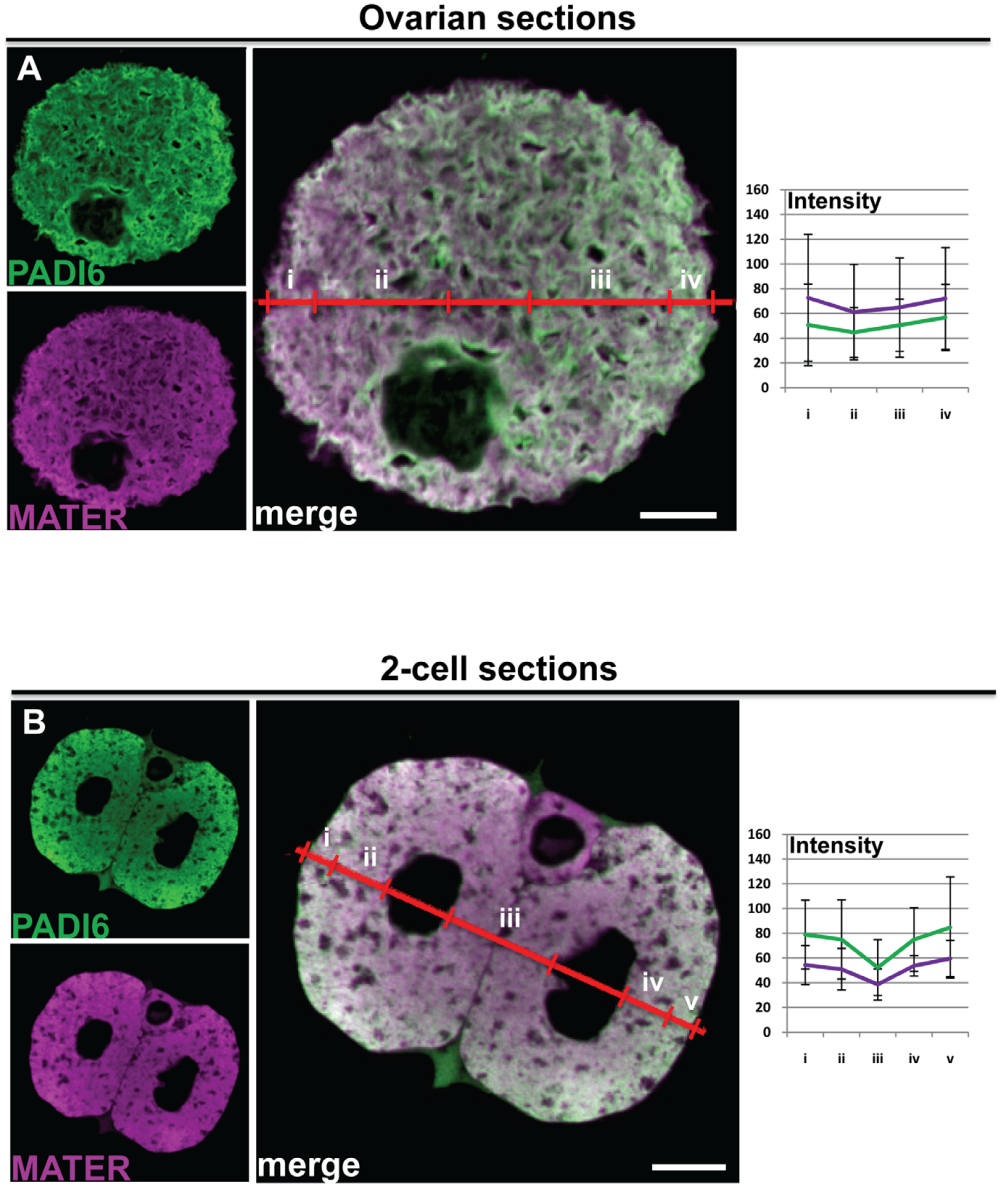
IF of cross sections reveal that PADI6 and MATER are primarily localized to the cytoplasm. Ovaries and oviducts were fixed, embedded in paraffin and sectioned before staining for IF. GV oocytes (A) and two-cell embryos (B) in the sections were visualized. PADI6 is shown in green and MATER in magenta. Included are graphs of the averages and standard deviations of four intensity profiles of four images per condition of the different stains in either cortical (i and iv) or cytosolic (ii and iii) zones for the oocytes or cortical (i and v), basolateral cytosolic (iii) or apical cytosolic (ii and iv) zones for the embryos. Bars, 10 µm.

After establishing that, in some situations, stronger fixation conditions enhanced the cytoplasmic versus cortex staining patterns, we next decided to investigate PADI6 and MATER expression in embedded ovarian and oviduct cross sections with the idea that by both fixing and embedding the oocytes, CPL-associated proteins would remain fixed in their correct positions. Further, by probing an oocyte cross section, as opposed to an intact whole mount oocyte, we would also rule out the possibility that the signal at the cortex was due to the anti-PADI6 and MATER antibodies binding to factors at the oocyte cortex and thus not being able to penetrate deeper into the cytoplasm. Mice were injected accordingly as described above, and then the appropriate tissues were harvested, fixed in formalin, embedded, sectioned, placed on slides and processed for image analysis as described in the materials and methods. Results show that the staining pattern of PADI6 and MATER in these sections was remarkably robust throughout the cytoplasm of GV oocytes (fig. 4A) and two-cell embryos (fig. 4B) (as demonstrated by the average intensity profile graphs) and there are no statistical differences between cortical and cytoplasmic signal for either GV oocyte sections (PADI6: p = 0.618; MATER: p = 0.644) or 2-cell embryos (PADI6: p = 0.652; MATER: p = 0.504). This reduced cortical signal is especially evident when these images are compared with those in figure 1. In light of the fact that ethanol dehydration of paraffin-embedded tissue section can lead to tissue shrinkage, we also tested the localization of PADI6, MATER and MSY2 by cryosection analysis, which does not include a dehydration step. Results showed that the localization patterns of these factors in oocyte cryosections was similar to that seen in the embedded sections (Fig. S3). Importantly, however, while not statistically significant, the cortical signal for both PADI6 and MATER does appear to be slightly elevated in two-cell embryos when compared to that seen in oocytes. Note the intensity profile in Fig. 4B which shows that the PADI6 signal, and to a lesser degree, the MATER signal is lower on the basolateral side of each nucleus when compared to the apical side (compare intensities of section iii to ii or iv). Also note that as opposed to whole mount preparations, the elevated cortical signal seen in embryos from embedded sections is more graded and extends deeper into the cytoplasm. This observation suggests that there is a subtle shift in the localization of both MATER and PADI6 toward the cortex as the oocyte transitions into an early embryo.

### The CPL density is homogenous in GV oocytes but not in two-cell embryos

**Figure 5:**
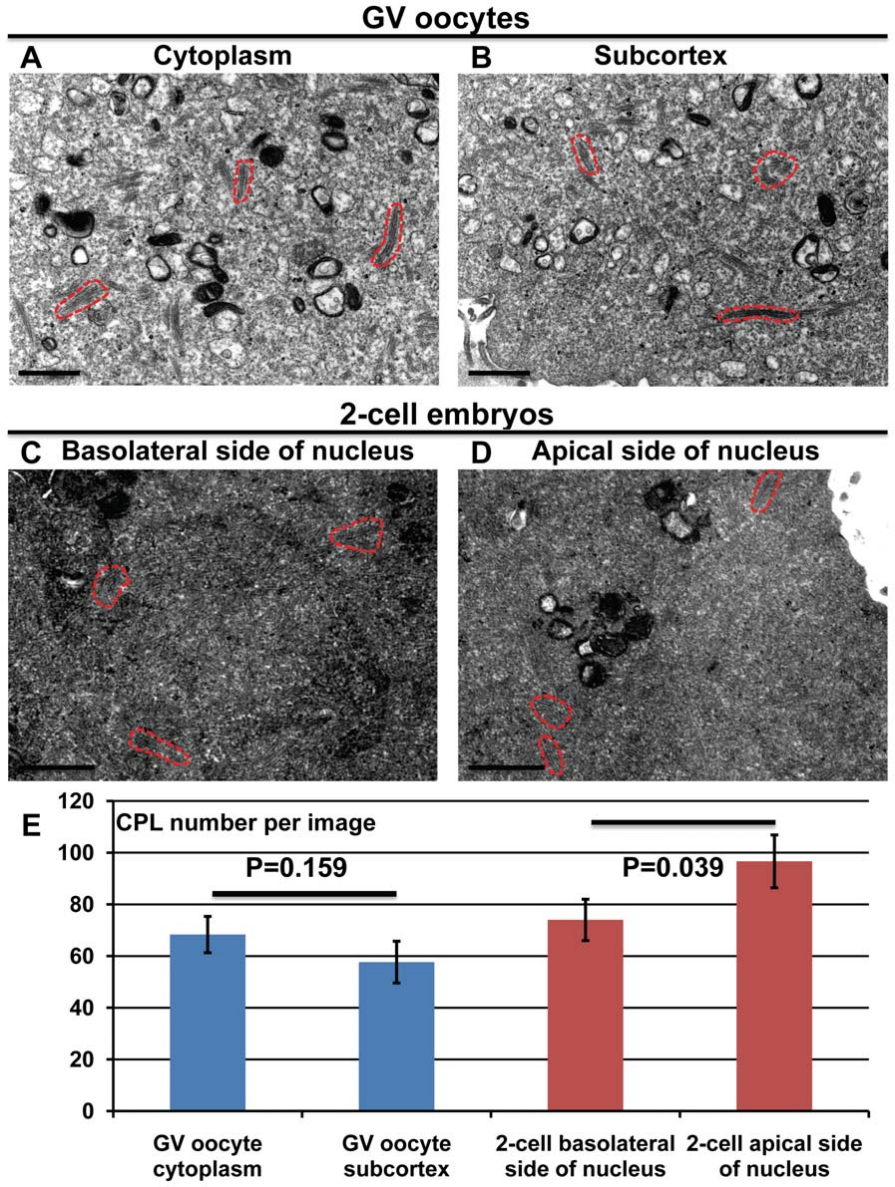
Quantification of CPLs in GV oocytes and two-cell embryos. GV oocytes (A and B) and two-cell embryos (C and D) were prepared for TEM. Representative images in the cytoplasm near the nucleus (A) or at the subcortex (B) of GV oocytes, and on either the basolateral (C) or apical (D) side of the nucleus in two-cell embryos. (E) The graph indicates the average number of CPLs per image in the four different aforementioned regions, as well as the two-tailed *t*-test P values. Representative CPLs are outined in dashed red lines. Bars, 1 µm.

We next sought to investigate the potential cause of this subtle shift in the confocal IF staining pattern for PADI6 and MATER from that of uniformly cytoplasmic in oocytes to a slightly more graded cortical signal in early embryos. Given that both PADI6 and MATER localize to the CPLs, we hypothesized that the increased subcortical IF signal in two-cell embryos may be due to increased lattice concentration in this region when compared to oocytes. To test this idea, we prepared GV oocytes and two-cell embryos for transmission electron microscopy (TEM) and the results are shown in figure 5. Images were taken in the cytoplasm (fig. 5A) and at the subcortex (fig. 5B) for GV oocytes, and in the basolateral (fig. 5C) and apical (fig. 5D) sides of each nucleus in two-cell embryos. We then selected images for each region and counted the CPLs visible in these images (fig. 5E). In the case of the GV ooyctes, surprisingly, there appeared to be more CPLs in the cytoplasm than in the subcortical region; although the median number of CPLs between the two regions was not statistically different (p = 0.159). However, analysis of CPL concentration in two-cell embryos found that there was a significant increase (p = 0.039) in the median number of CPLs found on the apical side of each blastomere nucleus when compared to the basolateral side. These findings support the hypothesis that the elevated apical confocal IF signal for both PADI6 and MATER in two-cell embryos is due to an increase in CPL density in these regions.

## Discussion

In this study, we have found that temperature and, particularly, time, of primary antibody incubation of whole mount oocyte and embryo preparations can strongly influence the localization of two CPL-associated proteins, PADI6 and MATER. Interestingly, the localization of MSY2, which does not appear to associate with the lattices (based on its lack of co-localization with PADI6), is only slightly affected by different incubation conditions, suggesting this phenomenon may be more restricted to CPL-associated proteins. The potential mechanism behind why PADI6 and MATER do not appear to be strongly “locked” into position following fixation, and thus migrate over time toward the oocyte subcortex remains unclear. One possibility is that fixation with 4% PFA for 30 minutes does not adequately cross-link the CPL superstructure itself and the lattices move towards the oocyte subcortex in a time-dependent manner. However, TEM analysis suggests that this is not the case as we did not see any strong movement of the lattices to the oocyte cortex following extended incubation periods (data not shown). Alternatively, it is also possible that these fixation conditions do not fix a more soluble subset of the PADI6 and MATER protein pool to the CPLs and thus, during the longer incubation periods, this subset dissociates from the CPLs and diffuses to the oocyte subcortex. To add to this idea, when oocytes are incubated O/N 4°C in IF buffer, and then stained 1 h RT with primary antibodies, the resulting images are very similar to those where the primary antibody was incubated O/N 4°C (fig. S4). This would seemingly indicate that longer incubation time alone is the cause of this “drifting”. However, when oocytes that were stained for 1 h RT and then placed in IF buffer O/N 4°C, the images look like the normal 1 h RT condition, suggesting that the antibodies themselves can further lock structures into place, if done at the correct temperature, perhaps by linking two different molecules via each paratope of one antibody, creating aggregates that would further enhance fixation in general. Again why these factors would accumulate at the cortex is not clear, although our Triton X-100 experiments would suggest that membrane permeability is not a factor. However, one could imagine that other factors at the cortex, such as microfilaments, could specifically bind and retain PADI6 and MATER.

Our finding that formalin fixation of whole mount oocytes allowed for better PADI6 cytoplasmic retention supports the hypothesis that 4% PFA treatment may not sufficiently fix CPL-associated proteins. This prediction is more strongly supported by the observation that PADI6 and MATER localization in cross sections of formalin fixed ovaries and oviducts is primarily limited to the cytoplasm of oocytes and early embryos. IF analysis of tissue cross sections involves fixation of tissues with a 10% formalin solution followed by paraffin embedding, dehydration with ethanol, sectioning, and staining of the tissues once they have been deposited on slides. In addition to the higher formalin concentration, which should further enhance protein cross-linking, the ethanol dehydration step also helps to fix proteins, thus enhancing cytological preservation [13]. Therefore, it stands to reason that this type of processing would result in a much stronger immobilization of PADI6 and MATER when compared to analysis by whole mount IF, in which the proteins that have been cross-linked by PFA fixation remain within the aqueous environment of the relatively large volume of the oocyte cytoplasm and thus may be subject to mechanical shear stresses. Given the above observations, and that we have previously shown by immuno-EM that PADI6 appears to primarily localize to the CPLs [1], we predict that the cross sectional confocal IF staining pattern for PADI6 represents the most accurate representation of PADI6′s cellular localization. Further, given our recent finding that MATER is also required for CPL formation [5] and that another group has recently demonstrated by immuno-EM that MATER localizes throughout the cytoplasm to the CPLs [6], we predict that MATER is also primarily cytoplasmically localized.

A more precise determination of PADI6 and MATER localization seems warranted at this time given that MATER has recently been described by other investigators as being a member of the Sub Cortical Maternal Complex (SCMC), which also contains the maternal factors, Filia, TLE6, and Floped. As the name implies, this complex appears to primarily lie just beneath the oocyte plasma membrane at the subcortex [4], [7], [14] and then becomes asymmetrically restricted to the apical cytocortex in two-cell embryos. Interestingly, another SCMC member, Floped, has been found by a different group to both localize to the oocyte CPLs throughout the cytoplasm and to be required for CPL formation [6]. Similar to PADI6 and MATER, the investigators also found by whole mount confocal IF, that Floped appears to primarily localize to the oocyte and early embryonic subcortex, which was in contrast to their immuno-EM findings. They then evaluated Floped localization on paraffin sections of oocytes and embryos and, similar to our findings here, found that the Floped signal was not enriched at the cortex but instead was primarily distributed throughout the cytoplasm.

The findings that at least two SCMC components are required for CPL formation suggest that, either components of SCMC play an indirect regulatory role in CPL formation or that the SCMC and CPLs actually represent the same structure. Given the findings reported here and the findings of others discussed above, we currently predict that the latter hypothesis is correct. The potential discrepancy between the cortical versus cytoplasmic localization for these factors may be partially resolved by the observation that, while the localization of PADI6 and MATER in paraffin sections of GV stage oocytes in this study was uniformly cytoplasmic, their localization does become slightly more cortical in the two-cell embryos and this cortical movement correlates with the shift of the lattices to the cortex following fertilization. Therefore, the apparent strong subcortical localization of the SCMC to the subcortex may actually be reflective of the shift of the CPLs towards the subcortex in early embryos.

In conclusion, our findings suggest that the time-dependent redistribution of PADI6 and MATER in whole mount preparations is likely due to the incomplete fixation and cross-linking of these factors during processing. While we currently do not understand why fixation of oocytes with 4% PFA does not completely cross-link CPL-associated proteins, we would recommend to future investigators who wish to study the localization of CPL-associated proteins by whole mount, that stronger fixation methods are utilized. Additionally, our findings also suggest that the localization of CPL-associated proteins may be best carried out using fixed and embedded paraffin cross sections of oocytes and embryos as opposed to whole mount. Lastly, these findings also support the hypothesis that the SCMC and CPLs may represent the same structure.

## Materials and Methods

### Ethics statement

Animals were bred and maintained in accordance with Cornell animal care guidelines. Our animal protocol (#2007-0113) was approved by the Cornell Institutional Animal Care and Use Committee before implementation.

### Collection and preparation of oocytes and preimplantation embryos

All germinal vesicle (GV) stage oocytes and two-cell embryos were collected from 5- to 8-week old CD1 mice, which were obtained from Jackson labs and bred in our facilities. GV stage oocytes were isolated from ovarian follicles ∼46 hours after pregnant mare serum gonadotropin (PMSG) (Calbiochem) (10 IU) stimulation and two-cell embryos were isolated from the oviducts of superovulated and mated female mice ∼42 hours after human chorionic gonadotropin (hCG) (Sigma-Aldrich) (10 IU) treatment. Oocytes and two-cell embryos were cultured in M2 Medium (Sigma-Aldrich) supplemented with Penicillin (20 IU/ml) and Streptomycin (20 ug/ml) (Cellgro), and 5 uM Milrinone (Sigma-Aldrich) for GV oocytes before processing for either IF, IHC or TEM.

### Immunofluoresence and scanning confocal microscopy

After collection, the oocytes and embryos were fixed in 4% formaldehyde (EM Sciences) in 0.1 M PBS final for 30 minutes. The oocytes and embryos were then washed in IF buffer (1% BSA, 0.5% normal goat serum in 0.1 M PBS final) three times for 5 minutes. They were then permeabilized with 0.5% Triton X-100 in 0.1 M PBS final for 30 minutes, followed by three 5 minute washes in IF buffer. Next, the ooyctes and embryos were incubated with primary antibodies, diluted in IF buffer for 1 hour at room temperature or overnight at 4°C, followed by three 5 minute washes in IF buffer. The oocytes were then incubated with the secondary antibodies, also diluted in IF buffer, for 1 hour at room temperature, followed by three 5 minute washes in IF buffer. Finally, the oocytes and embryos were incubated in Slowfade Gold antifade agent (Invitrogen) mounting medium for 1 hour at room temperature before being mounted between slide and coverslip and visualized with a 510 LSM laser scanning confocal microscope (Zeiss). Optical sections of 1 µm were obtained, and the imaging parameters were set using the greyscale function in the Zen software (Zeiss) so that all the images were as intense as possible but under the point of saturation. All oocytes or embryos in the same treatment group were visualized with the same confocal settings. Confocal settings were adjusted to each treatment group in order to guarantee the best possible images for each treatment group without saturation. The images were then exported as TIFF files and processed for final figure formatting.

### Immunohistochemistry

After collection, the tissues were fixed in 10% Neutral Buffered Formalin (EMD Chemicals) for 4 hours at room temperature and then transferred to 4°C overnight. The next day, they were embedded in paraffin, and 5 µm sections were obtained and placed on poly-L-lysine coated slides. The slides were then put into a Xylene bath for 5 minutes, drained, and put into two more additional Xylene baths for 5 minutes each. Following the Xylene baths, the slides were then rehydrated in successive decreasing concentration alcohol baths, from 100% to 95% to 70%, all for 5 minutes and then finally washed three times in PBS 0.1 M for 5 minutes per wash. For antigen retrieval, the slides were microwaved twice for 10 minutes in 10 mM Citrate buffer pH 6, and then washed three times 5 minutes in PBS 0.1 M. Blocking was performed with 10% normal goat serum in 2× casein (Vector Labs) applied for 20 minutes at room temperature in a humid chamber. The liquid was then blotted off the slides, and the primary antibodies (diluted in 1× casein PBS 0.1 M) were applied to the slides, which were then incubated 2 hours at 37°C. The slides were then washed four times in PBS 0.1 M, and the secondary antibodies (diluted in PBS 0.1 M) were added to the slides for 20 minutes at room temperature. Finally, the slides were washed with PBS 0.1 M three times for 5 minutes before being coverslipped with Hard Set mounting medium (Vector Labs) and visualized via confocal scanning microscopy (Zeiss).

### Transmission electron microscopy

Electron microscopy was performed as described elsewhere [3] with the following modifications. After collection, GV oocytes and two-cell embryos were immediately fixed with 2.5% Glutaraldehyde (EM Sciences), 4% PFA, 0.1% tannic acid and 0.01 M MgCl2 in 0.1 M sodium cacodylate buffer (pH 7.3) at room temperature for 2 hours then overnight at 4°C. The oocytes were post-fixed with 1% osmium tetroxide in cacodylate buffer for 1 hour, en bloc stained with 2% uranyl acetate, dehydrated in a graded ethanol series and then embedded in LX-112 resin (Ladd Research). Thin sections (50–70 nm) were cut with a diamond knife (Diatome) on an AO/Reichert ultramicrotome and picked up on nickel 200 mesh thin bar grids. Grids were contrast stained with 2% uranyl acetate followed by Sato's modified lead stain. Samples were examined by a FEI T12 TWIN transmission electron microscopy (TEM) at 100 kV and images were collected with a Gatan Orius® dual-scan CCD camera.

### Antibodies

Primary antibodies: Guinea Pig anti-PADI6, used at 1∶1,000 for IF and IHC, (Wright et al., 2003); Rabbit anti-MSY2, used at 1∶1,000 for IF and IHC, a kind gift from Richard Schultz, UPENN; Rabbit anti-MATER, used at 1∶1,000 for IF and IHC, a kind gift from Lawrence Nelson, NICHD. Secondary antibodies: Goat anti-guinea pig antibodies coupled to AlexaFluor 488, used at 1∶200 for IF and IHC (Invitrogen); Goat anti-rabbit antibodies coupled to AlexaFluor 546, used at 1∶200 for IF and IHC (Invitrogen).

### Statistical Analysis

For the IF image analysis, a minimum of ten oocytes or embryos were treated per condition in each experiment and all of the oocytes or embryos demonstrated very similar staining patterns dependent on the condition.

For the statistical analysis of IF images, four different random images from each condition were analyzed using the profile intensity function of the Zen confocal software (Zeiss). For each intensity profile, cortical and cytoplasmic regions (referred to in the figures as i and iv, or ii and iii, respectively) were defined based on the distance from the plasma membrane and nucleus, as well as signal intensity, where appropriate, using the graphs obtained. After defining each region, all of the data points in each region were averaged for each image, and then averages of each region between all four images were generated, using Microsoft Excel 2007. Standard deviations were also calculated four the averages generated for all four images. Statistical significance was calculated using a two-tailed *t*-test in the Microsoft Excel 2007 program by comparing the average values obtained in sections i and iv to ii and iii in the case of oocytes, and comparing sections i and v to ii and iv for 2-cell embryos. P-values<0.05 were regarded as statistically significant.

For the TEM image analysis, three different random images were analyzed from the region in the cytoplasm near the nucleus (cytoplasm) or just under the cytoplasmic membrane (subcortex) for the GV oocytes, and from the region between the nucleus and the apical cytoplasmic membrane (apical side) or between the nucleus and the cytoplasmic membrane interface of the two blastomeres (basolateral side) for the two-cell embryos. Means and standard error of the mean (SEM) were calculated for the number of CPLs in each image and statistical significance was calculated using a two-tailed *t*-test in the Microsoft Excel 2007 program. P-values<0.05 were regarded as statistically significant.

## Supporting Information

Figure S1 **Time influences the staining pattern of PADI6 and MATER more than temperature.** GV oocytes were prepared for IF and stained with antibodies against PADI6 (A-D), MATER (A and B) or MSY2 (C and D). Primary antibody incubation was carried out at either 1h 4°C (A and C) or O/N RT (B and D). PADI6 is shown in green, MATER in magenta and MSY2 in red. Bars, 10µm.
(TIF)

Figure S2 **The staining patterns of lipid droplets and tubulin are not affected in the different antibody incubation conditions.** GV oocytes were prepared for IF and stained with Nile Red (5 µg/ml) (A and B) and antibodies against PADI6 (A-D) or against alpha-tubulin (1:1000, Sigma-Aldrich, T5168) (C and D). Primary antibody incubation was carried out at either 1h RT (A and C) or O/N 4°C (B and D). PADI6 is shown in green, Nile Red and alpha-tubulin in red. Bars, 10µm.
(TIF)

Figure S3 **Oocyte cryosections show similar staining patterns to the paraffin-embedded sections.** Ovaries were extracted and frozen in OCT before sectioning and staining with antibodies against PADI6 (A and B) and MATER (A) or MSY2 (B). PADI6 is shown in green, MATER in magenta and MSY2 in red. Bars, 10µm.
(TIF)

Figure S4 **Overnight incubation in IF buffer followed by primary antibody staining for 1h RT shows staining patterns similar to the primary antibody O/N 4°C incubation condition.** GV oocytes were prepared for IF and stained with antibodies against PADI6 and MSY2. Primary antibody incubation was carried out at either 1h RT (A), O/N 4°C (B), for 1h RT after an overnight incubation at 4°C in IF buffer (C) or 1h RT before an overnight incubation at 4°C in IF buffer (D). PADI6 is shown in green and MSY2 in red. Bars, 10µm.
(TIF)
