# Supplementary figures and images for: Subcellular Localization of Cytoplasmic Lattice-Associated Proteins Is Dependent upon Fixation and Processing Procedures
Source: PLoS One. 2011 Feb 16;6(2):e17226. doi: 10.1371/journal.pone.0017226 (PMC3040232; doi:10.1371/journal.pone.0017226)

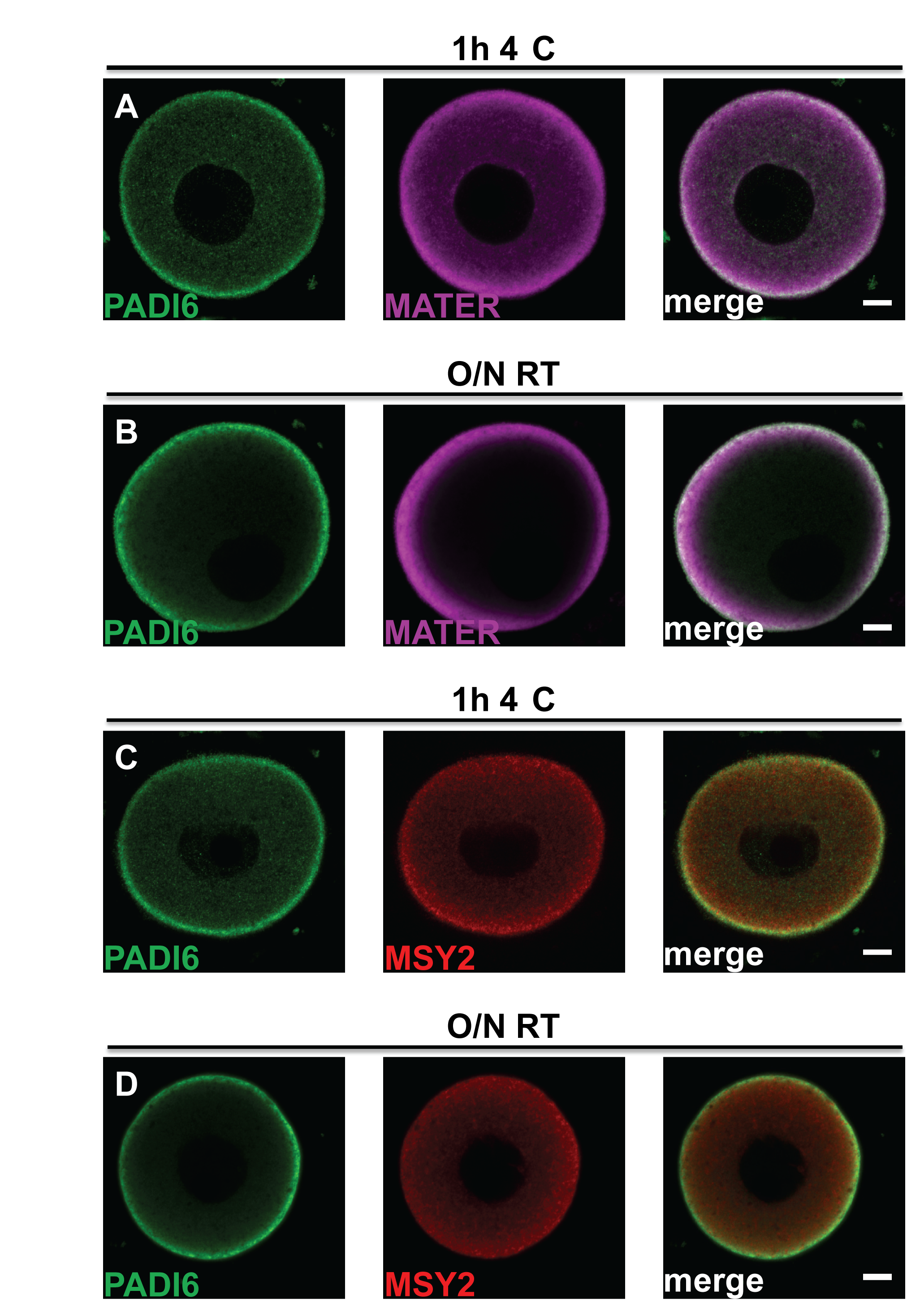

Supplement: Figure S1 — Time influences the staining pattern of PADI6 and MATER more than temperature. GV oocytes were prepared for IF and stained with antibodies against PADI6 (A-D), MATER (A and B) or MSY2 (C and D). Primary antibody incubation was carried out at either 1h 4°C (A and C) or O/N RT (B and D). PADI6 is shown in green, MATER in magenta and MSY2 in red. Bars, 10µm. (TIF) [file pone.0017226.s001.tif]

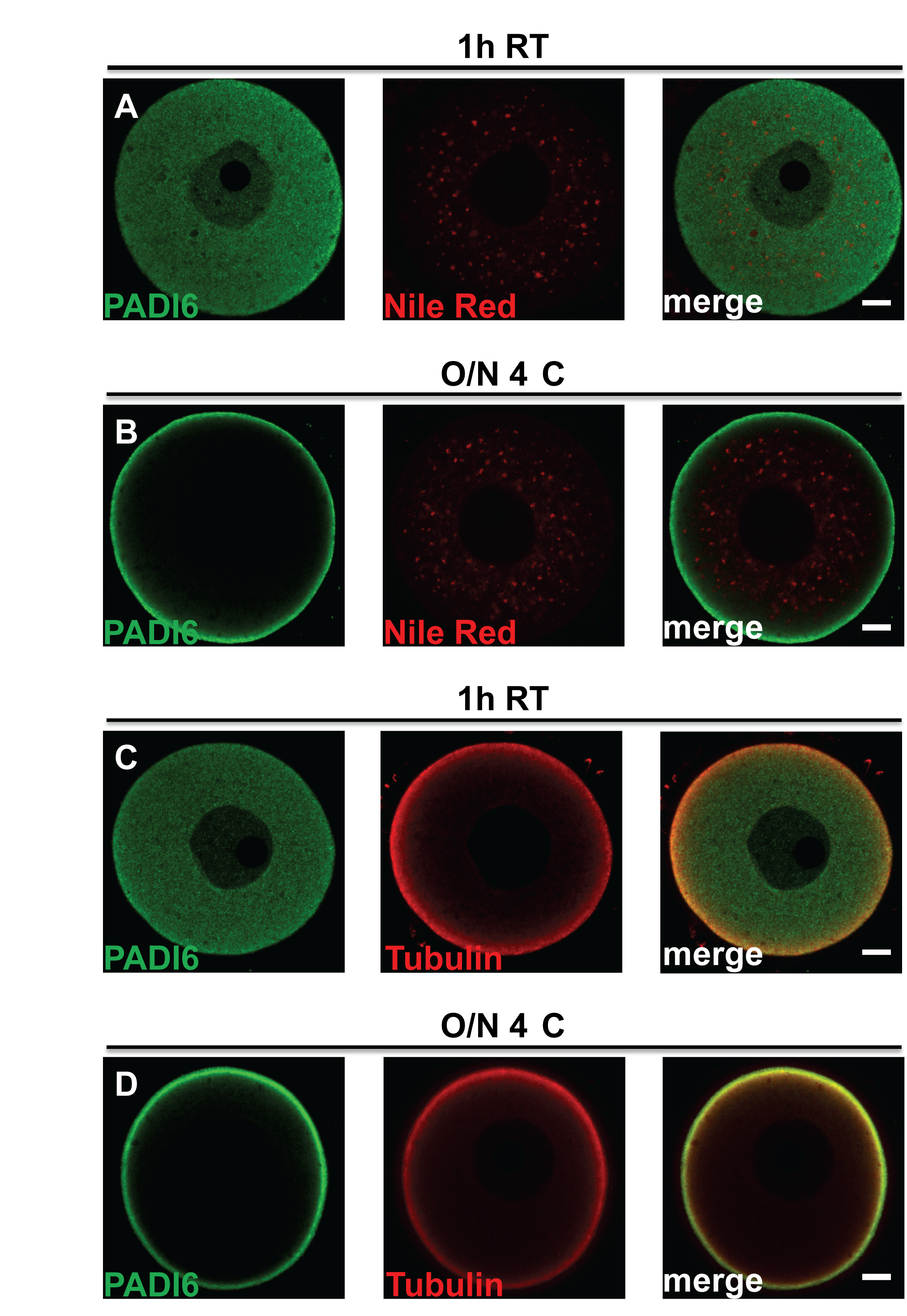

Supplement: Figure S2 — The staining patterns of lipid droplets and tubulin are not affected in the different antibody incubation conditions. GV oocytes were prepared for IF and stained with Nile Red (5 µg/ml) (A and B) and antibodies against PADI6 (A-D) or against alpha-tubulin (1:1000, Sigma-Aldrich, T5168) (C and D). Primary antibody incubation was carried out at either 1h RT (A and C) or O/N 4°C (B and D). PADI6 is shown in green, Nile Red and alpha-tubulin in red. Bars, 10µm. (TIF) [file pone.0017226.s002.tif]

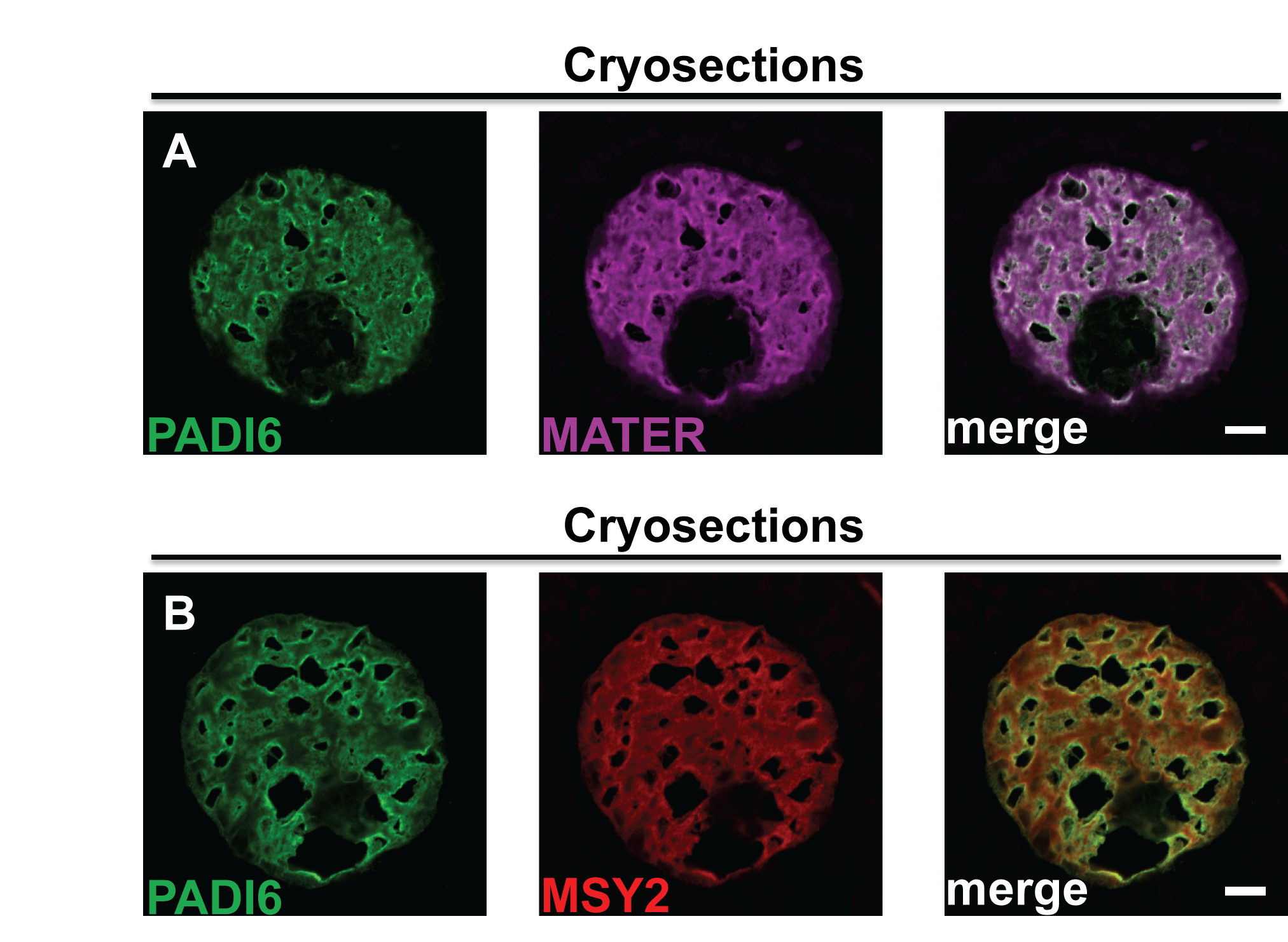

Supplement: Figure S3 — Oocyte cryosections show similar staining patterns to the paraffin-embedded sections. Ovaries were extracted and frozen in OCT before sectioning and staining with antibodies against PADI6 (A and B) and MATER (A) or MSY2 (B). PADI6 is shown in green, MATER in magenta and MSY2 in red. Bars, 10µm. (TIF) [file pone.0017226.s003.tif]

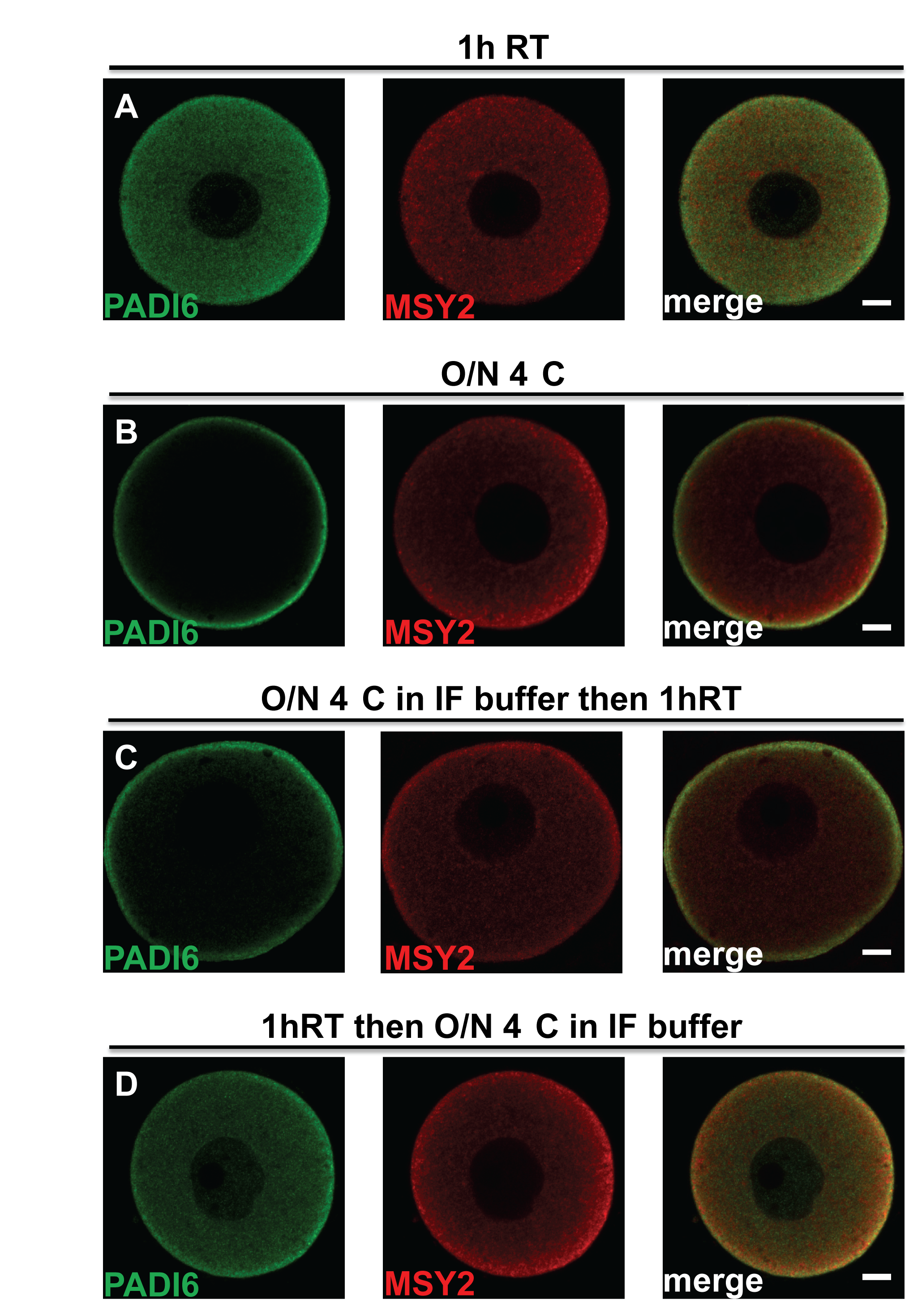

Supplement: Figure S4 — Overnight incubation in IF buffer followed by primary antibody staining for 1h RT shows staining patterns similar to the primary antibody O/N 4°C incubation condition. GV oocytes were prepared for IF and stained with antibodies against PADI6 and MSY2. Primary antibody incubation was carried out at either 1h RT (A), O/N 4°C (B), for 1h RT after an overnight incubation at 4°C in IF buffer (C) or 1h RT before an overnight incubation at 4°C in IF buffer (D). PADI6 is shown in green and MSY2 in red. Bars, 10µm. (TIF) [file pone.0017226.s004.tif]
